# An Efficient Methodology for Brain MRI Classification Based on DWT and Convolutional Neural Network

**DOI:** 10.3390/s21227480

**Published:** 2021-11-10

**Authors:** Muhammad Fayaz, Nurlan Torokeldiev, Samat Turdumamatov, Muhammad Shuaib Qureshi, Muhammad Bilal Qureshi, Jeonghwan Gwak

**Affiliations:** 1 Department of Computer Science, University of Central Asia, 310 Lenin Street, Naryn 722918, Kyrgyzstan; muhammad.fayaz@ucentralasia.org (M.F.); muhammad.qureshi@ucentralasia.org (M.S.Q.); 2 Department of Mathematics and Natural Sciences, University of Central Asia, Khorog 736, Tajikistan; nurlan.torokeldiev@ucentralasia.org; 3 Department of Mathematics and Natural Sciences, University of Central Asia, 310 Lenin Street, Naryn 722918, Kyrgyzstan; samat.turdumamatov@ucentralasia.org; 4 Department of Computer Science and IT, University of Lakki Marwat, Lakki Marwat 28420, KPK, Pakistan; muhdbilal.qureshi@gmail.com; 5 Department of Software, Korea National University of Transportation, Chungju 27469, Korea; 6 Department of Biomedical Engineering, Korea National University of Transportation, Chungju 27469, Korea; 7 Department of AI Robotics Engineering, Korea National University of Transportation, Chungju 27469, Korea; 8 Department of IT & Energy Convergence (BK21 FOUR), Korea National University of Transportation, Chungju 27469, Korea

**Keywords:** classification, convolutional neural network, discrete wavelet transform, MRI

## Abstract

In this paper, a model based on discrete wavelet transform and convolutional neural network for brain MR image classification has been proposed. The proposed model is comprised of three main stages, namely preprocessing, feature extraction, and classification. In the preprocessing, the median filter has been applied to remove salt-and-pepper noise from the brain MRI images. In the discrete wavelet transform, discrete Harr wavelet transform has been used. In the proposed model, 3-level Harr wavelet decomposition has been applied on the images to remove low-level detail and reduce the size of the images. Next, the convolutional neural network has been used for classifying the brain MR images into normal and abnormal. The convolutional neural network is also a prevalent classification method and has been widely used in different areas. In this study, the convolutional neural network has been used for brain MRI classification. The proposed methodology has been applied to the standard dataset, and for performance evaluation, we have used different performance evaluation measures. The results indicate that the proposed method provides good results with 99% accuracy. The proposed method results are then presented for comparison with some state-of-the-art algorithms where simply the proposed method outperforms the counterpart algorithms. The proposed model has been developed to be used for practical applications.

## 1. Introduction

With billions of neuronal cells, the human brain presents one of the intricate patterns of structural and neural connectivity in the human organism. The characterization of different sets of the brain, for instance, led to a new multidisciplinary approach in the study of networks [1,2,3]. The brain connectivity characterizes networks of brain regions connected by anatomical traits [4,5]. Understanding biological neuronal networks, particularly in the human brain, requires proper knowledge of the network architecture of the whole brain [6]. Thus, over the past decades, the brain-mapping methods and neuroimaging techniques for the pattern of neuronal networks gained great interest [7]. In this context, the wide range of quantitative analysis of imaging datasets in the study of the human brain plays an essential role in detecting brain disorders and early diseases [8].

Magnetic Resonance Imaging (MRI) is a medical imaging modality that attracts attention in biomedical engineering and is known as a safe, non-invasive, non-persistent, and pain-free diagnostic technique. Medical images can be obtained from X-ray radiography, Computed tomography (CT), and other modalities. However, Magnetic Resonance Imaging (MRI) does not cause radiation and employs a uniform magnetic field and Radiofrequency (RF) to expose the human body to gain images of the internal body system. MRI images can be presented with high-quality images in terms of resolution and contrast in 3D and 2D formats. These digital formats give a vast amount of information about internal diseases for soft tissue differentiation and further analysis and classification. MRI can provide detailed information about abnormalities in the soft tissue [9] that may not be determined by CT or X-ray radiography.

In this paper, we proposed a new method based on convolutional neural network (CNN) and discreate wavelet transform for brain MRI classification. Recent advances in neuroimaging techniques have resulted from complex neurological disorders, often in terms of several challenges in early diagnosis and treatment. On the one hand, these developments have taken place due to continuously produced medical data thanks to tangible progress in automated CAD systems in medical imaging informatics. MRI-based medical images, for instance, are more than pictures; they are data [10,11]. On the other hand, most biomedical images show differences in brightness, shape, and texture [12]. Due to its intrinsic nature, the segmentation process of any medical image is a time-consuming and challenging task [13]. Therefore, as the images of the human brain fall together with ‘big data’ [14], there is an increasing demand for an automated image processing to analyze and classify in terms of the latest applications in machine-learning techniques [15].

Deep learning (DL) is a subfield of machine learning that extends traditional neural network (NN) to models that mainly focus on feature learning. Compared to other DL models, a CNN with a set of algorithms and techniques has become a successful tool in MRI image classification [16]. An important aspect of CNN in DL is that the necessary features can be learned through directly providing images known as end-to-end strategy, i.e., there is no need to extract information from images first to feed CNN [17]. CNN has three fundamental mechanisms: a local receptive field, weight sharing, and subsampling, and consists of several layers, including convolutional and pooling layers, and each feature map in a pooling layer is connected to a feature map in a convolutional layer. CNN has been widely used in medical imaging for breast tissue classification and lung nodule detections. Later CNN became very popular in the MR image classification for tumor-like lesions and tissue segmentation and detection and deep cortical and subcortical white matter structures and tissue segmentation [18].

Quantitative analysis of MRI-based images, in general, plays a vital role in clinical diagnosis for the treatment of neurological diseases. With a high resolution, MRI easily detects signals emitted from normal and abnormal tissue [19], providing valuable information in distinguishing healthy and diseased brains. Several studies previously have examined in developing machine learning algorithms for MRI-based image segmentation of normal (e.g., white and gray matter) and abnormal brain tissues (e.g., brain tumor) [13,15,20,21]. Nevertheless, the classification of brain MRI slices as normal and abnormal is still a challenging task [21]. Developing a robust segmentation method, for instance, is a crucial element in the successful classification of brain MRI images [13]. This paper deals with the novel classification of MRI data of normal and pathological brain tissues using a robust segmentation method that employs deep learning technique based CNNs. In recent years, CNNs have gained significant interest in medical imaging [22,23] and have become more prevalent in image classification methodology.

In image analysis methods, feature extraction is a method of dimension reduction. At some point, the following process concentrates on the extraction of specific features from brain MRI images [24]. Several methods reported different techniques for feature extraction in image classification, wavelet transform-based techniques [25] such as Discrete Wavelet Transform (DWT) [26] and Continues Wavelet Transform (CWT) [27]. For feature reduction studies, the most used techniques are already available, e.g., Linear Discriminate Analysis (LDA) and Genetic Algorithm (GA) [28], Independent Component Analysis (ICA) and Principal Component Analysis (PCA) [29]. Wavelets transform, for instance, has become a prevalent choice for multiple imaging techniques and MRI classification features, thanks to its effective non-stationary signal analysis method [30]. In this context, we have proposed a novel approach for image classification by integrating wavelet transform to extract features from MRIs.

This work has proposed a novel method based on Discrete Wavelet Transform (DWT) and Convolution Neural Network (CNN) for brain MRI classification. The main contribution is the new assembling of a discrete wavelet transform with the convolutional neural network. The discrete wavelet transform has been used to remove unnecessary detail and make the image more informative and efficient for machine learning algorithms to classify. The reason behind using discrete wavelet transform with a convolutional neural network is that the approximate images returned by discrete wavelet transform have denser information and proficiency for classification than original images.

Most research focused on the classification of brain images as normal or abnormal for abnormal brain MRI studies. In the presence of any pathological appearance, the next stage will be location identification and the medical recommendation. The division of brain MR images into normal and abnormal can be carried out in two ways: (1) using the conventional machine learning models, e.g., artificial neural network, logistic regression, k-nearest neighbors, decision tree, support vector machine, and random forest; (2) using deep learning models, e.g., CNN, stacked autoencoder (SAE), Boltzmann machine (BM), long short-term memory (LSTM), etc. The conventional classification models and the deep learning models have their pros and cons when applied for image classification.

When the normal or standard classifiers are used for the purpose, the major contribution is the feature extraction stage, in which a minimal representation of an image is fed as input to the classifier. In this architecture, some well-known features of images are extracted, reduced, and then given as the input to the classifier. The major drawback associated with this phenomenon is the loss of information during the feature extraction and feature reduction stage. On the other hand, if the feature is not extracted from the image or not even reduced, the classifier is not too powerful to perform the processing of the whole image or a higher number of features efficiently. Hence, a trade-off is required for gaining sufficient information from the images. Eventually, the number of features extracted must neither be too high nor too low for maintaining a fruitful outcome.

Similarly, if deep network models are used to classify brain MRI images, the whole image is given as input to the model for performing the classification task. To process the whole image, the deep network models developed are highly complicated. The complex models add extra processing time and effort to the model processing. In all previous works where deep models have been used for classification, the authors have used the whole image as input to the model, resulting in more processing time as outlined. This drawback of deep models can be overcome if, instead of the whole image, another representation of an image with a smaller size is given as input to the deep model.

Our contribution is three-fold: Firstly, the identification of representation of image adequately enough to represent the whole image without any information loss. After extensive experimentation, the final and summarized representation was the Harr wavelet which is more effective and the simplest wavelet in the wavelet’s family. Other wavelets were also included in the experiments, but their information possessing capability cannot maintain the information. Secondly, compared to previously proposed CNN or other deep models, our approach has a simple CNN architecture due to the reasons mentioned above. Eventually, our proposed model provides simplicity as compared to other deep models with significantly added performance.

The rest of the paper is organized as follows: Section 2 presents a brief review of the related work. Section 3 briefly describes the method implemented in this paper. Section 4 is carried out with the implementation, experimental results, and discussion. Lastly, the conclusion part is presented in Section 5. The abbreviations with their corresponding descriptions are listed in Table 1.

## 2. Related Work

Over the past decades, several studies reported on computer-based neuroimaging techniques for characterization and processing of MRI brain images that have become the tool of choice for the diagnosis of brain disorders and early treatment [10,24,31,32,33,34,35,36]. At the same time, however, automated segmentation and classification of normal and pathological brain structures are one of the most challenging tasks [15]. Nonetheless, numerous approaches have been developed applying machine learning techniques to detect the structural, functional alterations in the human brain; some are described in this section. For instance, the classification of MRI data in image processing is often a costly, laborious, and time-consuming task [37].

Numerous works have been done toward feature extraction, segmentation, and classification of MRI images to develop different versions of algorithms and deep learning models. Many authors have used conventional techniques integrated with modified algorithms for the preprocessing of MRI images, then following the steps of computer-aided diagnosis (CAD) frameworks to urge the ultimate outputs. All endeavors were aimed at the best models to extend the performance of brain image classification. Many authors applied DWT feature extraction tools to feed a neural network model for MRI classification for image feature extraction purposes. For instance, Chaplot et al. 2006 [25] employed a DWT feature extraction as an input to ANN and support vector machine (SVM) for brain disorder detection, and Maitra et al. [38] presented two-stage algorithms of orthogonal DWT for feature extraction and SVM for image classification. Kumar et al. 2017 [39] proposed a slightly different model, where authors used DWT feature extraction, genetic algorithm principal component analysis (PCA), and SVM classification. PCA was implemented to reduce the number of features, and this hybrid method aimed at MRI tumor classification. El-Dahshan et al. 2010 [40] used DWT for feature selection and forward back-propagation artificial neural network (FP-ANN) and k nearest neighbor (KNN) classifier tools for MRI brain image classification. A method of clustering Fuzzy C-means (FCM) was utilized by Mohsen et al. 2018 [41] to image segmentation, and they used DWT for feature extraction and deep neural network for MRI brain tumor classification.

High accuracies in brain MRI classification have been achieved by Wahid et al. [42], who proposed a method based on statistical moments and probabilistic techniques. Statistical moments have been employed for feature extraction, and ANN has been used for feature reduction. Zahid et al. [43] proposed another methodology for brain MRI classification using DWT, color moments, and ANN. The DWT method has been used for image decomposition and removed low detail from the image to obtain an approximate small-sized image. A Harr wavelet of three levels of decomposition has been applied to the images. The first three statistical moments are then calculated for each channel and total of 9 features are obtained that have then been further fed to ANN for classification. Slightly different combination was proposed by Amin et al. [44]. They presented an MRI tumor classification that employs DWT-based image fusion with Daubechies kernel, a global thresholding method for segmenting tumor region and CNN model. A 23 layered CNN architecture utilizes convolutional, batch normalization, rectified linear activation unit (ReLU), down sampling through max pooling, fully connected network, and final output layer softmax to classify normal and pathological brain structures.

Masood et al. [45] has proposed a method based on fuzzy logic and convolutional neural network for brain tumor detection. In the preprocessed step the image enhancement is used for image segmentation, fuzzy logic has been used for edge detection, and convolutional neural network has been used to classify the brain images into meningioma and non-meningioma. The proposed method is compared with some well-known methods and the results indicate that the proposed method performed well as compared to counterpart algorithms. Muzammil et al. [46] proposed for improved clinical diagnosis using an innovative multimodal image fusion technique. Obdusami et al. [47] suggested a method for mild cognitive impairment (MCI), late mild cognitive impairment (LMCI), early mild cognitive impairment (EMCI), and Alzheimer’s diseases (AD) prediction using the finetuned ResNet18 network. The results exhibit that the accuracy of this method is high as compared to conventional methods. A similar approach for pathological brain detection has been proposed by Zhang et al. [48] based on three components namely wavelet packet Tsallis entropy, extreme learning machine, and java algorithm. It was noted that the proposed method outshines the existing methods.

To make the abnormal image classification process more efficient, Jude et al. 2019 [49] used simple assignment processes rather than the weight adjustment process to reduce computational complexities in conventional CNN architecture. Utilizing the 2D CNN approach of Simonyan and Zisserman [50], Kamnitsas et al. [51] presented a more discriminative 3D CNN model and processed multi-scale parallel convolutional pathways for MRI brain tumor segmentation particularly for large data sets. Pereira et al. [52] presented their own CNN architecture with the same approach, which employs small, cascaded kernel layers rather than single and bigger ones. This model benefits from fewer weights of the network and results in an effective MRI image segmentation. To discriminate small lesions (<1.5 cc), Liu et al. [53] proposed a modified version of the CNN algorithm, where authors employed one more sub-path to Kamnitsas et al. CNN model [51] for the MRI brain metastases segmentation process. To increase classification execution, Togacar et al. [54] proposed the recursive feature elimination (RFE) embedded CNN model enhanced with hypercolumn technique and supported by networks like AlexNet and VGG-16, and SVM classifiers. Inspired by the residual neural networks, Remedios et al. [14] presented a 3D CNN architecture for MRI contrast classification and named their model as PhiNet designed for specific diseases like Alzheimer’s, sclerosis, and traumatic injuries.

In addition, some authors presented hybrid models to outperform traditional deep learning techniques. For example, Cinar et al. 2020 [55] presented a hybrid CNN architecture, which is a sophisticated and modified version of the original Resnet50 CNN model [56] to increase the accuracy rate. The development occurred by removing the last 5 layers of Resnet 50 and adding 10 different layers. Khan et al. [57] proposed a hybrid model developed by cascading support vector machine with three pathway CNN models. A different hybrid model approach was presented by Kruthika et al. [58] for MRI Alzheimer segmentation and classification. This hybrid model consists of fast learning capsule networks (3D CapsNet), 3D autoencoder, and 3D CNN. Chang et al. [59] proposed a combined CNN model and conditional random fields (CRF) to increase MRI brain image segmentation accuracy. This two-pathway CNN model employs max and min pooling layers on each. Finally, a comparative approach was proposed by Talo et al. [60]; they compared the following well-known trained CNN models: ResNet50, ResNet35, ResNet18, AlexNet, and VGG 16 to classify MRI images into normal and pathological brain structures, i.e., inflammatory, neoplastic, degenerative and cerebrovascular categories using Harvard Medical school MR image datasets. Different methods along with strengths and weakness have given below in Table 2.

The current studies have some limitations in one way or another way; some methods are good in accuracy but take a lot of time to compute. Some are fast, but the accuracy of these algorithms is poor. Hence, there is extensive to develop model to tackle these issues.

## 3. Proposed Methodology

**Figure 1:**
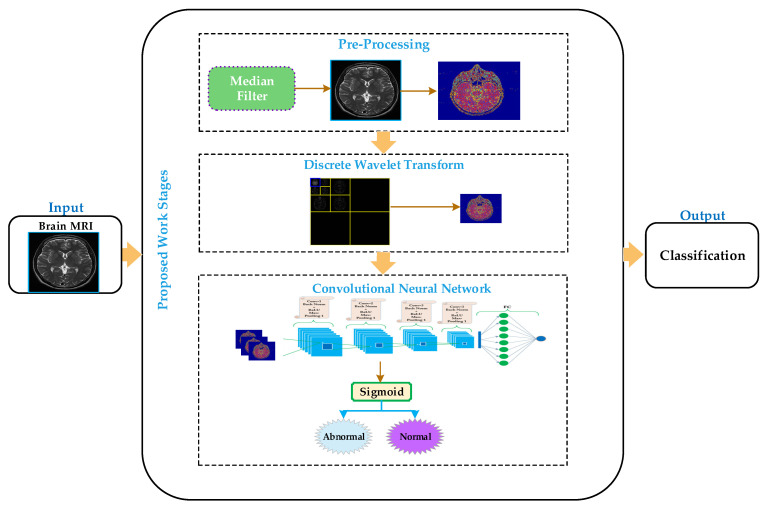
Processing diagram of the proposed model for brain MRI classification.

The proposed methodology carried out the discrimination of MR images into normal or abnormal. The proposed approach comprises four stages: preprocessing, feature extraction, classification, and visualization, as depicted in Figure 1. In the preprocessing stage, the median filter has been applied to enhance the image’s quality remove salt and pepper noise. In the feature extraction stage, the discrete wavelet transform has been applied on the smoothed image to obtain a small size approximate image by removing some unnecessary and unrelated information from the original image. We have applied the Harr wavelet in the proposed work, which is more effective and the simplest wavelet in the wavelet’s family. In the classification stage, the convolutional neural network has been applied to classify the brain MRI into normal or abnormal. The convolutional neural network approximates images as inputs from the discrete wavelet transform and classifies them into normal or abnormal in the classification stage.

### 3.1. Preprocessing

**Figure 2:**
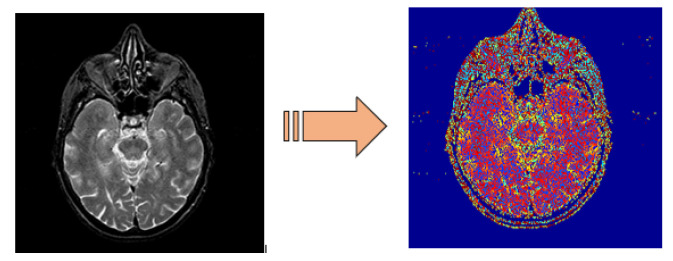
Normal image (**left**) and RGB image (**right**).

Numerous types of image filters are available in the literature, such as mean filter, median filter, wiener filter, and different types of image noises that exist, such as space noise, Gaussian noise, salt and pepper noise, speckle noise. Hence, it is of utmost importance to apply an appropriate filter for noise removal for images. In the proposed work, we have used the MR grayscale brain images, and these types of images are affected by salt-and-pepper noise [42,61]. For this purpose, we have used a median filter to remove salt-and-pepper noise from MR brain images. The median filter has the capability of sharpening the images and preserving the edges. We have used window size: 3 × 3 masks; this size is suitable because the large window size of the mask affects the image edges and requires high computation time. After salt and paper removal the grayscale images are converted to RGB images, Figure 2 shows a grayscale image along with RGB image. These RGB images are further fed as inputs to the DWT layer to get an approximate image size.

### 3.2. Discrete Wavelet Transform

As mentioned earlier, we employed discrete wavelet transform to extract the approximate image. Wavelet transform uses a windowing technique with variable size; thus, it preserves both the time and frequency information of the signal. The main advantage of the wavelet is the adoption of scale instead of adopting frequency, in short, the DWT produces a time-scale view rather than a time-frequency view. The timescale is an efficient and powerful way of viewing the data [62].

**Figure 3:**
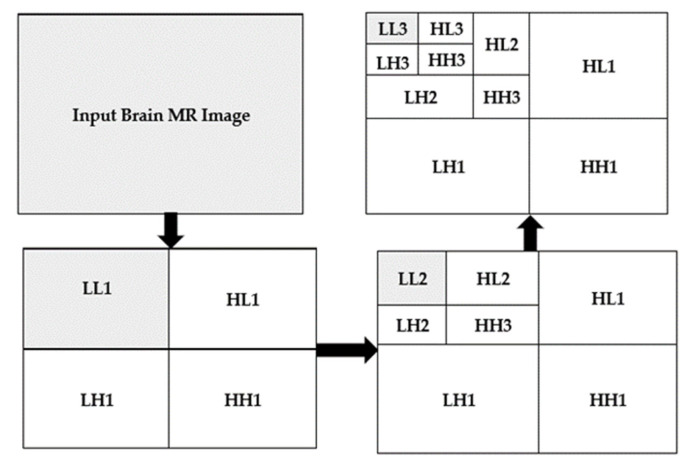
2D three-level decomposition method.

In this work, the focus is on 2D imaging; hence, it is required to use DWT to each dimension disjointedly. The schematic diagram of the DWT is illustrated in Figure 3. As depicted, that each level is divided into four sub-bands, namely Low-Low (LL), Low-High (LH), High-High (HH), and High-Low (HL) images at each level, and 1, 2, and 3 signify the according levels. The division of the LL sub-band is further carried out into the four previous stated bands. The detailed component of the image is represented by LH, HL, and HH [63]. The compaction of the image is incremented with the incrementation of levels, but the quality of the desired approximate image is decreasing. In this work, a level-3 decomposition was carried out using Harr wavelet to approximate solid information. In the first level of decomposition, the image is divided into four sub-bands, namely LL1, LH1, HH1, HL1, in which the LL represented approximate image (LL1) which is of essential and further it is considered for processing. In the second level of decomposition, the LL1 is further decomposed into four sub-bands, namely LL2, LH2, HH2, and HL2. The LL2 approximate image is further considered for processing. In the third level of decomposition, the LL2 is further decomposed in four sub-bands named LL3, LH3, HH3, and HL3.

### 3.3. Convolutional Neural Networks (CNNs)

Methods based on deep learning usually provide better results than shallow learning-based methods, i.e., classical dense artificial networks [44]. In the proposed work, the convolutional neural network has been considered for brain MRI classicization. The convolutional neural network nowadays is extensively used for classification purposes in different areas.

We applied a convolutional neural network on 64 × 64 × 3 approximate images obtained through DWT. Our CNNs architecture has six types of layers with different parameters. They are convolutional layer, batch normalization, ReLU, max pooling, fully connected, and Sigmoid. Images that have been used in the proposed work are 2D images; hence, 2D convolutional layers have been applied to the input images. For image normalization, the batch normalization filter has been used by applying fg1 and variance $\sigma_{B}{}^{2}$ over mini-batch. The $B_{n}$ activations are evaluated by using the Equations (1)–(3).

(1) $$B_{n}=\frac{fg_{1\;\left(x,\;y\right)-\mu_{B}}}{\sqrt{\sigma_{B}{}^{2\;}+\varepsilon }}$$

In Equation (1) ε indicates the stability factor. The stability factor is used for stability improvement in the case of smaller batch sizes.

(2) $$B_{n}=\tilde{\gamma }N_{a}+\tilde{\beta }$$

In Equation (2) the parameters $\tilde{\gamma }$ and $\tilde{\beta }$ represent the offset and scale factors, respectively. The $\tilde{\gamma }$ and $\tilde{\beta }$ factors are updated during the network training process. ReLU is used to perfom thresholding operations as given in Equation (3).

(3) $$f\left(x\right)=\{\begin{array}{c} -x,\;x\geq 0\\ x,\;x<0 \end{array}$$

The down sampling is performed through the max-pooling layer. There is a connection between all preceding layers £ neurons, and all features are combined that are learned through the previous layer. A sigmoid layer is used to classify based on probability given in Equation (4).

(4) $$\psi \left(£\right)=\frac{exp\;a_{r}}{\sum_{i=0}^{n}expa_{j}£}$$

**Figure 4:**
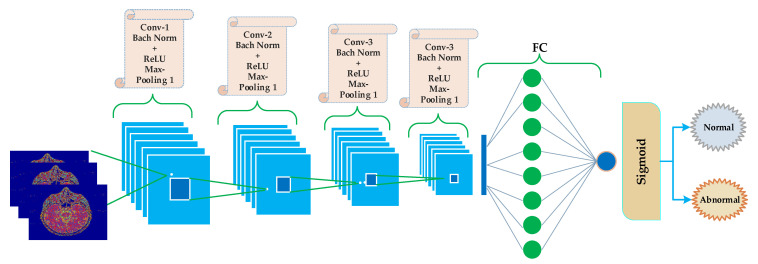
Proposed schematic CNN diagram for classification of brain MRI as normal and abnormal.

The proposed CNN structure consists of five blocks, as shown in Figure 4. The first four blocks are the same, holding convolution, batch normalization, ReLU, and max-pooling layers. The last (5th) block is different from the previous four blocks. The fifth layer contains the FC layer following by a sigmoid layer.

**Figure 5:**
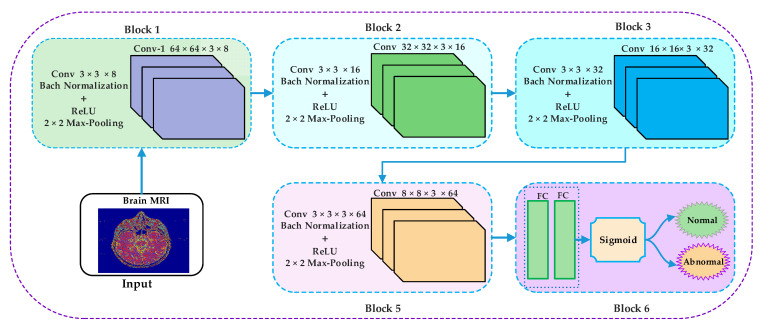
Block-wise architecture of the proposed model.

The block-wise architecture of the proposed model is illustrated in Figure 5. The kernel size of convolutional layers in the first four blocks is represented using Equation (5).

(5) h×w×c 

where *h*, *w*, and *c* represent the height, width, and channel, respectively. The *h* = *w* = 3 in the first four blocks, *c* = 8 in the first block, *c* = 16 in the second block, *c* = 32 in the third block, and *c* = 64 in the fourth block of the proposed model of CNN. A 2 × 2 max-pooling has been used in the first four blocks with stride = 2. Bach normalization has been applied to every block with 8, 16, 32, 64 channels.

## 4. Implementation, Results, and Explanations

### 4.1. Implementation Setup

This section briefly explains the implementation environment for the research and development of brain tumor detection from brain MRI images using CNN. All the implementation of the proposed work has been carried out on Intel(R) Core (TM) i7-7500U having NVIDIA GeForce 940MX GPU, 15 GB DDR2 RAM, and Window 10 is installed on it. In this study, we have built a convolutional neural network that is trained on brain MRI images. The convolutional neural network can predict and classify brain images as normal or abnormal. Graphics Processing Units (GPU) can significantly increase the training process of different models. Intensive computation, matrix multiplication, and other operations are involved in training models like image classification. We have used GPUs with machine learning frameworks to train the model in our experiment. In this study, TensorFlow and different libraries such as Keras, NumPy, and SciPy are used to build the convolutional neural network. For some graphical representations, Matlab 2019 has also been used.

**Figure 6:**
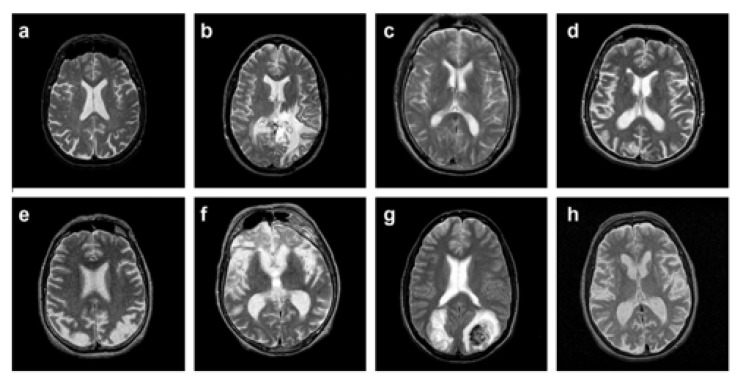
(**a**) normal brain MRI, (**b**) glioma, (**c**) meningioma, (**d**) Alzheimer, (**e**) Alzheimer plus (**f**) visual agnosia pic’s disease, (**g**) sarcoma, and (**h**) Huntington’s.

The dataset used in the proposed work is comprised of T2-weighted MR brain images in the axial plane and 512 × 512 in the plane resolution. The data are downloaded from [64]. 5000 images have been selected randomly, in which 2055 are normal, and 2045 are abnormal. The abnormal brain MR images comprise the below diseases: glioma, meningioma, Alzheimer, Alzheimer plus visual agnosia pic’s disease, sarcoma, and Huntington’s. A sample image from each disease is illustrated in Figure 6, along with a normal brain MR image.

Using the above dataset and implementation software tools, we have implemented each stage of the proposed work as discussed below. In the preprocessing, we have applied the median filter with (3, 3) size; the median filter has been chosen to remove salt and pepper noise because the MRI images are generally affected by such noises.

**Figure 7:**
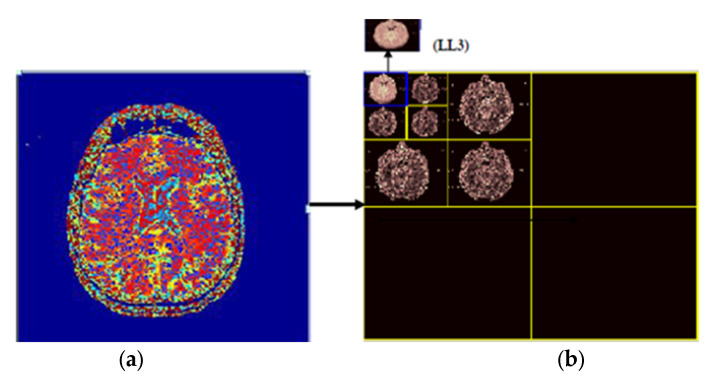
(**a**) original image, (**b**) 3 levels decomposition by using Harr wavelet.

The implemented 3 levels decomposition by using Harr wavelet is depicted in Figure 7. By using Harr wavelet, a three-level decomposition has been done, which significantly minimizes the input image size. The desired small-size approximate image after three decomposition levels is shown in the top left corner of Figure 7. The original images are of size 512 × 512 × 3, and after removing the low-level detail from the original image by using 3-level Harr wavelet decomposition, the size of the images is reduced to 64 × 64 × 3. In the proposed work, we have considered 3-level decomposition because this is an appropriate size; more decomposition levels increase the possibility of losing useful information from images.

In the proposed work, convolutional has been used on the input by using a convolutional filter for generating a feature map. A kernel of size (3 × 3) has been used as a convolutional filter, and the stride (2,2) is used to move the convolutional filter at each step.

**Figure 8:**
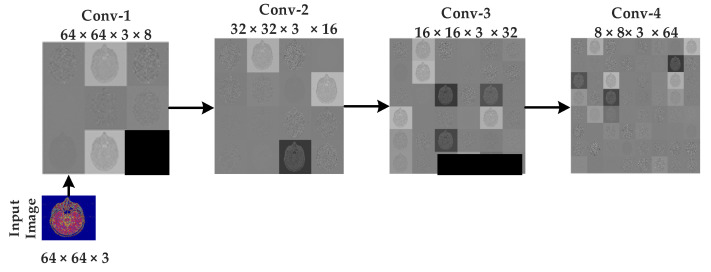
Intermediate results of sequences at CNN.

The approximate images achieved after applying 3 levels of decomposition using Harr wavelet are then fed to CNN algorithms for classification. As discussed earlier, the original images are of size 512 × 512 × 3 but after 3 levels of decomposition, the size is reduced, and 64 × 64 ×3 approximate images are achieved, which are further fed to CNN algorithms. 64 × 64 images are then rotated in different angles for the best learning rate before feeding to the CNN algorithm. The CNN algorithm takes the 64 × 64 approximate images as input, and eight different mean filters have been applied to images in the convolutional layer of the first block of the proposed model, as shown in Figure 8. In the second convolutional layer of the second block, 16 mean filters are convoluted on the features map of the first block after applying the ReLU activation function, batch normalization, and a (2,2) max pooling.

Similarly, in the third block, 32 mean filters are convoluted on feature maps of block 2 after applying the ReLU activation function, batch normalization, and a (2, 2) max-pooling. Hence in the results, the number of images generated is 32 of size 16 × 16 × 3. 16 × 16 × 3 sizes still huge image size, and if it is directly given to a fully connected layer, then computation time will be very high; hence we need to shrink the image further. Hence, in the fourth layer, we convoluted 64 mean filters of size (3, 3) on the second convolutional layer features map. In the fourth convolutional layer, the number of images is 64 with size 8 × 8 × 3. At this point, we felt that the 8 × 8 × 3 is an appropriate size for a fully connected layer. The 8 × 8 × 3 is then converted to a one-dimensional array and then fed to a fully connected layer. These features are then mapped to a fully connected layer. In the last layer, the sigmoid function has been used to classify the brain MR images into normal and abnormal.

In this study we have converted the grayscale images to color (RGB) images in order to get more information for binary image classification. From previous studies we have learnt that feature extracted are more informative as compared to grayscale Images [65]. The DWT has been used in this study to reduce the dimensionality, because the original images that we are feeding to the convolutional neural network are large in size (512 × 512 × 3), so the total number pixels per image are 786,432. In order to reduce the dimensionality of these images we have used discrete wavelet transform (DWT) to reduce the size of the images and obtained small size of images without losing useful information. In this work, a 3-level decomposition DWT has been used; it has significantly reduced the size of images to 64 × 64 × 3. Hence, feeding these small sizes of images greatly reduces the computational complexity of convolutional neural network. All networks are listed with their properties in Table 3.

**Figure 9:**
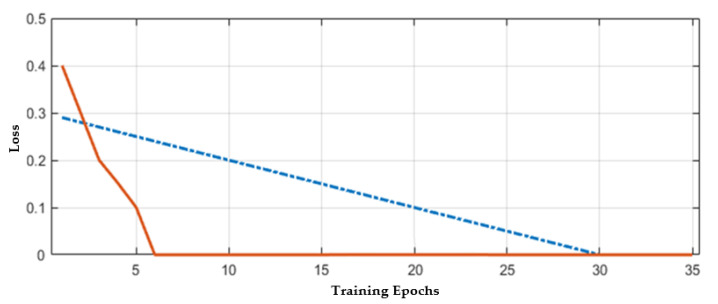
Accuracy with respect to loss.

The CNN model’s training is first carried out on a given dataset with trial and test error methods because there is no proper mechanism for an appropriate number of layers selection. Hence, the different number of layers (6, 10, 15, 20, 25, and 30) have been tried to obtain the best results, but when the number of layers increases or decreases from 25, then validation error is increased. Hence, in the proposed CNN model, 25 layers have been selected for further experiments. The hyperparameters castoff in our methodology is illustrated in Table 4. The stability of the network mostly occurs after 30 epochs; hence the total number of epochs that have been chosen here is 40 to train the model effectively. The validation errors are given in Table 5. In the proposed work, 70% of data have been selected to train the network, and 30% has been used for testing. The loss rate of the proposed model is shown in Figure 9. The proposed work binary classification has been performed to classify the brain MR images into normal or abnormal. In Figure 9, the training epochs are denoted by the x-axis and the y-axis denoting error or loss rate. The best prediction scores for training and VEs are obtained, indicating a reciprocal relationship between losses and accuracy.

### 4.2. Results and Discussion

For this, we have applied the convolutional neural network for brain MRI classification. There are many classifiers, such as support vector machine, k-nearest neighbor, artificial neural network, random forest, and we have applied these classifiers in our previous studies [24,42,66]. However, a convolutional neural network performs the best in the sense of accuracy in this study. Different authors have applied different techniques to the same set of data for brain MRI classification, but the results are not prominent. The overall accuracy of the results of the proposed method is determined by using the performance evaluation factors, such as Kappa statistics, which is a measurement metric that carried out the comparison of the observed accuracy with the expected accuracy. This is considered true positive (TP), which indicates the accurate prediction of the positive class, false positive (FP), which indicates the inaccurate prediction of the positive class [24,42]. The precision is a performance matric that uses TP and FP factors to define the degree of measurements given in Equation (6).

(6) $$\mathrm{Precision}=\frac{\mathrm{TP}}{\mathrm{TP}+\mathrm{FP}}$$

Another performance measurement is Recall which is a fraction of the total amount of relevant rederived instances, given in Equation (7).

(7) $$\mathrm{Recall}=\frac{\mathrm{TP}}{\mathrm{TP}+\mathrm{FN}}$$

where false negative represents the accurate prediction of negative class.

The receiver operating chrematistic curve (ROC) is a graphical plot equating the TP and the FP rates of a classifier. These mentioned measures values are given in tables and figures for performance measurement of the proposed model for brain MRI classification [67].

**Figure 10:**
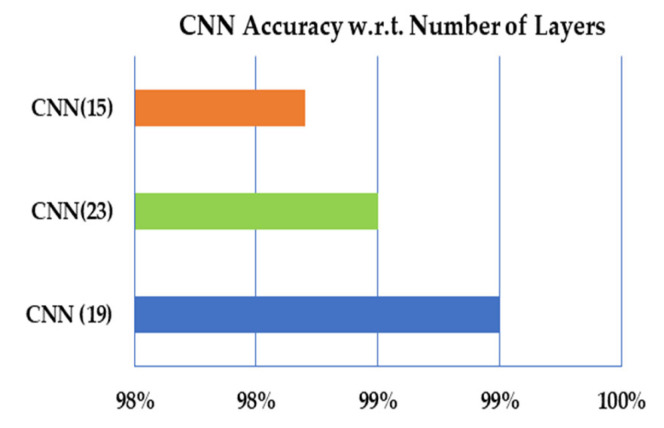
The overall accuracy of CNN with respect to the number of layers.

In the proposed work, we have calculated the performance measures to measure the proposed approach’s performance with different aspects. There is no proposer mechanism for defining the number of layers in the convolutional neural. In the proposed work, the trial and test have been defined to define suitable number layers for the proposed CNN model. For each number of layers, the performance measures have been calculated, and the results exhibit that the model with 19 layers performs better than 23–15 layers, as illustrated in Table 6 and Figure 10, respectively.

**Figure 11:**
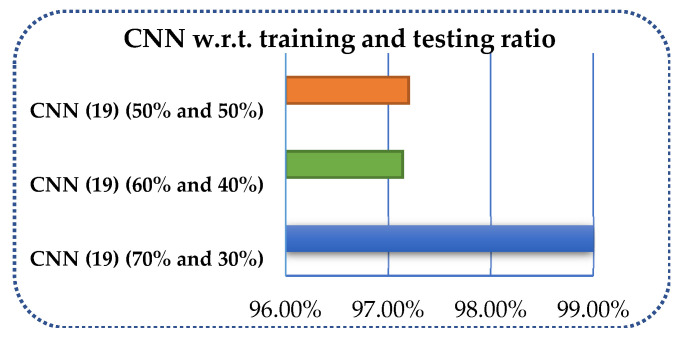
Overall accuracy of CNN with respect to training and testing ratios.

Secondly, despite the accurate number of layer specifications, the measurement of the proposed method has been carried out using different amounts of data for training and testing. The data have been divided into 70 and 30%, 60 and 40%, and 50 and 50% in the proposed CNN model, and performance measures have been calculated for each splitting as shown in Table 7 and Figure 11.

**Figure 12:**
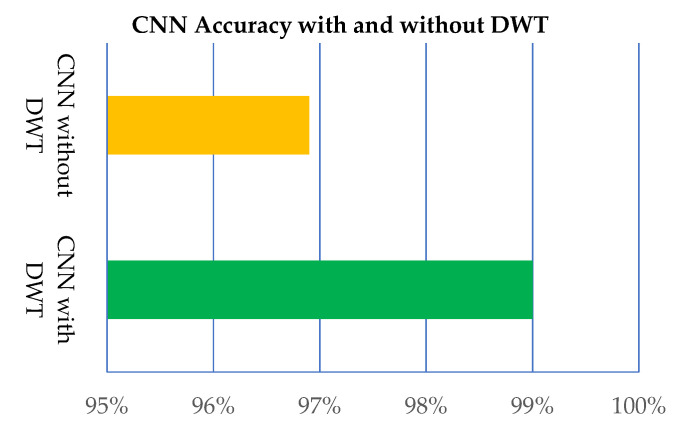
Performance of the proposed CNN model with and without DWT.

Further, the proposed model is applied without DWT and with DWT, as shown in Table 8 and Figure 12. The results provided by the proposed CNN model with DWT are better than the CNN without DWT.

The comparison of the proposed method is carried out with some well-known brain MRI classification methods. In all these methods, the same number of images and same modality (MRI) images have been used, and the results illustrate that the proposed method is far better as far better in terms of training accuracy, testing accuracy, minimum training loss, and minimum testing loss compared to the counterpart methods. The proposed method accuracy results with some state-of-the-art techniques have been listed in Table 9.

In this work, we have proposed a brain MRI image classification model comprised of preprocessing, discrete wavelet transform, and convolutional neural network. The MRI images are typically affected by salt-and-pepper noise [43,61]. To be able to remove noise, we employed an efficient mean filter. The discrete wavelet transforms, particularly Haar wavelet, have been used extensively to reduce the size of the images by applying different decomposition levels. We have used 3-level Harr wavelet decomposition to remove low-level detail from images and make them suitable for classification. The original 512 × 512 × 3 size images have been reduced to 64 × 64 × 3 by applying 3-level decomposition. Only images with high information have been fed to CNN to achieve high classification results. This model of CNN has been used extensively in image classification problems [44]. The proposed architecture has 19 layers: one input layer and four convolutional layers with 8, 16, 32, and 64 filters. The network has four normalization layers, two pooling layers of size 2 × 2 with a stride of 2. The CNN model also has four ReLU layers, one fully connected layer, a sigmoid layer for classification, and an output layer. The proposed model has been applied on MRI images taken from [68], where 5000 images with 2045 abnormal and 2055 normal images have been selected for experiments. The ROC method has been used for performance evaluation, and the proposed model has given 99% accuracy, which is quite prominent. The results gained from this model are compared with some well-known methods for brain MR image classification. The performance of the proposed model is far better in comparison to the counterpart approaches for brain MR image classification.

The proposed work is simple and efficient and provides good results, but the problem is that we can compare but there are some certain limitations to the proposed work. We have applied the proposed model to a comparatively large dataset, the proposed work performance will be poor on the small dataset. Another limitation of the proposed work is that we have applied the proposed to 2D images and have not checked feasibility on 3D images. We have also used only MRI images and have not considered CT images, or the fusion of both. In real life it is very difficult to get brain MRI images from hospital, because normally these images are private, and doctors hesitate to provide these images for experiments. Therefore, we have applied the proposed model on a large dataset.

## 5. Conclusions and Future Work

In this present method, discrete wavelet transform has been used with the convolutional neural network for brain MRI classification. Usually, we directly reduce the size of images when we provide data to the convolutional neural network, and it may cause the loss of important information. Hence, in the proposed model, we have used the discrete wavelet transform to reduce the size of images without losing any information. These reduced images are then fed to the convolutional neural network for classification. The proposed method has been evaluated on different performance evaluation metrics, and the results exhibit that the proposed model provides good results. The purpose of designing the proposed brain MR image classification model is to improve the performance of the current methods for brain MR image classification and provide an easy way for the radiologist to take measures. It is worth mentioning that the proposed model performs very well, and the accuracy of the proposed method is almost 100%. The proposed method has been evaluated in different aspects and different ways, and the results are prominent.

In the future, we would like to extend our model to multiple class classification as the current system provides only binary classification. We also want to evaluate our model on other modalities, e.g., PET and CT images. We will also design and a web-based interface to facilitate radiologists to use the system efficiently.

## Figures and Tables

**Table 1:** Notations with the descriptions.

Notation	Description
CNN	Convolutional Neural Network
DWT	Discrete Wavelet Transform
CT	Computed tomography
MRI	Magnetic Resonance Imaging
ReLU	Rectified linear unit
Cov	Convolution
LL	Low-Low Level Detail
HL	High-Low Level Detail
LH	Low-High Detail
HH	High-High Level Detail
FC	Fully Connected
TP	True Positive
TN	True Negative
PF	False Positive
ROC	Receiver Operating Characteristic
VEs	Validation Errors

**Table 2:** Different models with their strengths and limitations.

Reference	Model	Contribution	Limitation
[14]	Integrated model of CNN and Transfer Learning	Good classification accuracy on test data.	Very large and complex CNN model
[16]	Novel 3D CNN Method	Robust when training on one dataset and testing on another dataset.	Only designed for 3D images, and low classification accuracy
[36]	Autoencoder Deep Neural Network (ADNN)	Accuracy was improved	High Computation Complexity
[37]	Enhanced Approach using Residual Networks	High accuracy was achieved by considering small dataset.	Poor results on large dataset
[49]	Modified Deep Convolutional Neural Network	Computation complexity was reduced	Low classification accuracy
[60]	AlexNet, Vgg-16, ResNet18, ResNet34, and ResNet50	Used to classify five classes (normal, cerebrovascular, neoplastic, degenerative, and inflammatory)	Low classification accuracy
[61]	Color Moments and artificial neural network	Simple and very fast	Low classification accuracy
[43]	DWT, color moments, and artificial neural network	High accuracy	Good only on small dataset

**Table 3:** All network layers are listed with their properties.

Number	Layer Name	Layer Properties
1	Images (Input)	Size = 64 × 64 × 3
2	Conv-1	Convolutional (64 × 64 × 3 × 8) with stride 2
3	Bach Norm	Bach Normalization Operation
4	ReLU	Rectified Linear Unit
5	Max Pooling	Max-Pooling Operation (2 × 2, stride [2,2], padding = [same])
6	Dropout	50% dropout
7	Conv-2	Convolutional (32 × 32 × 3 × 16) with stride 2
8	Bach Norm	Bach Normalization Operation
9	ReLU	Rectified Linear Unit
10	Max Pooling	Max-Pooling Operation (2 × 2, stride [2,2])
11	Dropout	50% dropout
12	Conv-3	Convolutional (16 × 16 × 3 × 32, stride 2, padding = [0,0,0,0])
13	Bach Norm	Bach Normalization Operation
14	ReLU	Rectified Linear Unit
15	Max Pooling	Max-Pooling Operation (2 × 2, stride [2,2], padding = [same])
16	Dropout	50%
17	Conv-4	Convolutional (8 × 8 × 3 × 64) with stride 2
18	Bach Norm	Bach Normalization Operation
19	ReLU	Rectified Linear Unit
20	Max Pooling	Max-Pooling Operation (2 × 2, stride [2,2], padding = [same])
21	Dropout	50%
22	Fully Connected	512 hidden neurons in first hidden layer and 1024 in second hidden layer
23	Functions	tanh on first and second hidden layers neurons, and sigmoid on the output layer neuron.
24	Classification	Output (Normal or abnormal)
25	Loss	Binary Cross-entropy

**Table 4:** Hyperparameters of the proposed CNN model.

Max Epochs	Validity Frequency	Learning Rate
35	31	0.001

**Table 5:** Validation error for layers selection in the proposed CNN model.

Total Number of CNN Layers	Validation Error
6	0.12125
10	0.11358
20	0.10889
25	0.08000
30	0.09835
35	0.10486

**Table 6:** Overall accuracy of CNN with respect to the number of layers.

CNN (No. Layers)	Kappa Statistics	TP Rate	FP Rate	AUC	Recall	Precision
CNN (19)	0.9880	0.990	0.0013	0.9970	0.9970	0.9980
CNN (23)	0.9820	0.9850	0.0030	0.9990	0.9860	0.9880
SVM (15)	0.9780	0.9820	0.0040	0.9990	0.9804	0.9881

**Table 7:** Overall accuracy of CNN with respect to training and testing ratios.

Method (Ratio)	Kappa Statistics	TP Rate	FP Rate	ROC	Recall	Precision
CNN (19) (70 and 30%)	0.9880	0.990	0.0020	0.997	0.990	0.990
CNN (19) (60 and 40%)	0.93780	0.96150	0.03850	1	0.96150	97.14209
CNN (19) (50 and 50%)	0.96530	0.971	0.0060	0.9970	0.9710	0.9720

**Table 8:** Performance measurement of CNN with and without DWT.

CNN (No. Layers)	Kappa Statistics	TP Rate	FP Rate	ROC	Recall	Precision
CNN (19) with DWT	0.9880	0.99	0.0020	0.9970	0.99	0.99
CNN (19) without DWT	0.9627	0.969	0.0060	0.998	0.9690	0.9690

**Table 9:** Performance comparison in terms of accuracy and loss.

Model	Training Accuracy	Testing Accuracy	Training Minimum Loss	Testing Minimum Loss
CNNBCN-ER [61]	100.00%	94.85%	1.43 × 10^−3^	1.86 × 10^−1^
CNNBCN-WS [61]	100.00%	94.53%	6.61 × 10^−4^	2.20 × 10^−1^
CNNBCN-BA [61]	100.00%	94.53%	1.43 × 10^−3^	1.72 × 10^−1^
CNNBCN-ER1 [61]	100.00%	95.49%	1.07 × 10^−3^	1.69 × 10^−1^
CNNBCN-WS1 [61]	100.00%	95.17%	1.46 × 10^−3^	2.13 × 10^−1^
CNNBCN-BA1 [61]	100.00%	95.01%	1.13 × 10^−3^	1.71 × 10^−1^
Model 1 [68]	−	91.28%	−	−
Model 2 [67]	−	94.68%	−	−
Model 3 [69]	99.54%	94.20%	2.53 × 10^−2^	1.82 × 10^−1^
Model 4 [65]	−	94.39%	−	
Model 5 [70]	−	95.00%	−	−
Model 6 [71]	−	93.83%	−	−
Wide-Resnet-101 [72]	99.51%	91.14%	1.72 × 10^−2^	2.90 × 10^−1^
Mobilenet-v1 [73]	100.00%	93.88%	2.96 × 10^−3^	2.23 × 10^−1^
Proposed Method	100.00%	97.32%	3.42 × 10^−4^	1.53 × 10^−1^

CNNBCN-ER = Convolutional Neural Network Based on Complex Network—Erdos-Renyi. CNNBCN-WS = Convolutional Neural Network Based on Complex Network—Watts—Strogatz. CNNBCN-BA = Convolutional Neural Network Based on Complex Network—Baradasi-Albert. CNNBCN-ER1 = Modified CNNBCN-ER. CNNBCN-WS1 = Modified CNNBCN-WS CNNBCN-BA1 = Modified CNNBCN-BA.

## Data Availability

The dataset used in this work can be downloaded from the publicly available webpage: http://www.med.harvard.edu/AANLIB/home.html (accessed on 15 February 2021).
